# An Empirical Study on the Influence of Smart Home Interface Design on the Interaction Performance of the Elderly

**DOI:** 10.3390/ijerph19159105

**Published:** 2022-07-26

**Authors:** Chengmin Zhou, Yingyi Dai, Ting Huang, Hanxiao Zhao, Jake Kaner

**Affiliations:** 1 College of Furnishings and Industrial Design, Nanjing Forestry University, Nanjing 210037, China; yingyidai@njfu.edu.cn (Y.D.); tinghuang@njfu.edu.cn (T.H.); hanxiaozhao2022@163.com (H.Z.); 2 Jiangsu Co-Innovation Center of Efficient Processing and Utilization of Forest Resources, Nanjing 210037, China; 3 School of Art and Design, Nottingham Trent University, Nottingham NG1 4FQ, UK; jake.kaner@ntu.ac.uk

**Keywords:** smart home, interface design, interaction performance, age-friendly design

## Abstract

The concept of the smart home has been widely recognized and accepted, but the differentiated characteristics of elderly smart products in terms of demand and use are becoming more and more prominent. The lack of an efficient navigation design of the smart product interface increases the cognitive burden of elderly users, and how to better meet the needs of the elderly with smart products gradually becomes the focus of attention. This study was conducted for the elderly group, using the scenario-based design method to analyze the needs of elderly users, combining the research results of scenario theory with the smart home interaction design research method, focusing on how to make the style of interface navigation, sliding layout and button size more suitable for the cognitive behavior of elderly users. The purpose of this research is to realize an age-friendly smart home interaction design in terms of functional design and interface design. The experiment is divided into two stages: in stage 1, two different layouts and operation methods are commonly used for the age-friendly smart home interface: up and down sliding and left and right sliding; in stage 2, the functional buttons are square, where 4 styles are selected, and the side lengths are set to 10 mm, 12 mm, 15 mm, 18 mm and 22 mm. The sliding and retrieval test and retrieval and click test results show that for different sliding layout methods, the interactive performance and subjective evaluation of the interface with the up-and-down sliding layout are better. Among all functional button styles, the interaction performance and subjective evaluation of the simple button style with lines are the best. Among the function keys with a size of 10–22 mm, the interaction performance is better from 12 mm to 18 mm. The conclusion of the better interface data information obtained from this experiment improves the rationality of the age-friendly smart home interface and makes the smart home interface better for the age-friendly scenario.

## 1. Introduction

With the rise of the silver economy, the aging of the population, which affects the social and economic development of China, has gradually become a serious social problem that people are worried about, and the needs of the elderly are gradually brought to the attention of the market. The Ministry of Industry and Information Technology, the Ministry of Civil Affairs and the Health and Welfare Commission jointly released the “Action Plan for the Development of Intelligent Healthy Aging Industry”, which addresses the current situation of the increasingly rich and multi-level diversified aging needs of the elderly in modern society, and proposes to develop and innovate technologies, build data platforms and expand application scenarios to promote intelligent aging and healthy aging in China.

The research directions and contents of the foreign age-friendly smart home literature have focused on the following: research related to the use of sensors in smart solutions; implementation of remote access functions in smart home systems; research related to monitoring the elderly and manipulating devices through mobile rather than fixed devices; operational benefits of smart homes and output to smart scenarios; smart home design for specific characteristics of users research; and whether smart home design solutions have potential for direct application and the cost value of smart homes. Many foreign studies in this field involve smart furniture and home-related technologies, with a wide range of technologies and many in-depth and targeted studies, but the research content on age-friendly smart homes in China has limitations. Smart home research in China still focuses more on the technical aspects of smart home systems [1], and lacks analysis of the adaptability of the system to the user and the environment. Regarding the elderly, our researchers primarily focus on care and intelligent monitoring, exploring the use of smart homes to achieve remote monitoring or biometric-based identification, bed departure monitoring, fall monitoring, etc., focusing on the safety and security of elderly life. However, less attention has been paid to how to make the life of the elderly more convenient and comfortable, how to interact with or control the devices or homes when using smart homes, and emotional and humane care. A smart home can provide users with convenient, comfortable and safe daily life [2,3]. The services provided by smart homes bring various conveniences and efficient life, but the welfare enjoyers are mostly young groups, and the elderly are not able to use smart products without barriers due to their lifetimes, environmental factors and their behavioral characteristics and psychological features. In addition to the factors of elderly user characteristics, smart home products themselves also have the problem of emphasizing appearance over technology and neglecting user experience, with cumbersome and inhumane operations and too many functions but not enough practicality [4]. User experience and product usability are closely related to navigation design, the primary purpose of which is to help users find the desired information or complete the desired behavior or task. Efficient and straightforward navigation design guides users to the appropriate interface and leads to behavioral conversion [5]. Age-friendly smart homes as a product of Internet of Things (IoT) technology to be widely deployed face the same barriers that are the main issues that smart homes have—privacy, energy consumption, obstructiveness, usability, acceptability and cost [6,7]. Emphasis on age-friendly smart home design is considered to be a crucial means to improve the quality of life and ensure the health and safety of the elderly [8]. The General Office of the State Council issued a notice on the implementation plan to effectively solve the difficulties of using smart technology for the elderly, to let the elderly also enjoy the fruits of information technology development, starting from solving the difficulties encountered by the elderly when using smart technology so that the elderly can gain a sense of well-being in the information society. At present, the economic situation of the elderly in China and the national policy are very favorable to the development and application of smart home research, and in the future, age-friendly smart homes will develop in the direction of high practicality with flexible solutions, strong adaptability, low cost, ease of use, convenience and comfort.

In the field of interaction design, scenario interactive design (SID) focuses on how to focus on user behavior from different scenarios, and analyze the problems of the system based on user research results and update or rebuild new scenarios based on user value needs to achieve designs with usability [9,10]. Scenario-based design (SBD) refers to the user-centered design approach used in the early stages of the design cycle to capture the elements of human–product interaction through scenarios and stories. Rosson, Carroll et al. in 1998 described and analyzed the requirements in scenario-based design by considering the case studies development process [11]. In 2000, Carroll proposed five technical challenges addressed by scenario-based IT design [12]. In 2006, Robert Scoble convinced companies to embrace social media, then in 2013 proposed that social media, along with data, mobile devices, location technologies and sensors constitute the five converging forces, ushering in the era of scenarios. He discussed how technology can be the interface that connects people to their environment [13]. SBD refers to a user-centered design approach used in the early stages of the design cycle to capture the elements of human–product interaction through scenarios and stories [14]. HJ Kang in 2017 proposed smart services through four new dimensions, reflecting the characteristics of the smart service scenario framework, and also establishing an analytical framework for service experience based on traditional service scenarios and service blueprints [15]. In addition, SBD is increasingly being used in medical and clinical fields abroad, such as the study by Mancilla on adolescent well-being and mental health in 2015 [16] and Mohr on depression and anxiety in adults in 2017 [17]. With the construction and expansion of national open innovation platforms, the development of new technologies, such as artificial intelligence has broadened the boundaries of scenario applications, and the concept of scenarios has provided a new perspective for the development of smart homes and caused changes in their scenarios, where technology and scenarios are integrated to make them better serve people.

Therefore, the purpose of this study is to match the smart home interaction interface with age-friendly scenarios. The touch screen interface is the most direct and common interaction method for smart home devices, and it is also a more intuitive and easier way to support human–computer interaction [18]. In terms of recognizing commands and privacy, touch interaction has the highest accuracy rate compared to gesture and voice [19]. Furthermore, voice interaction has the problem of dialect, making it less practical. To explore the factors affecting the interaction performance of the elderly user group, we analyze the best form of data information to enhance the interaction performance and user preference in three aspects—sliding method [20], button size and button style [21,22]—to improve the interaction experience of elderly users using smart home and reduce the misoperation rate.

The experiment uses a scenario-based design approach, a smart scene panel as the interaction center, and a fusion of multimodal interaction to investigate both functional and visual perspectives based on the demand characteristics of elderly users [23]. Integrating scenario theory with user research, we obtain user behavior, pain points, needs, demand priorities and design opportunity points by building objective scenarios, and finally transform them into age-friendly smart home system functions and an age-friendly interaction interface design, verify the usability and age-appropriateness of the design by building test scenarios, discover the shortcomings of the design, and make improvement iterations. Most of the available smart home systems are targeted at young- and middle-aged users, and the interface design and function design are not targeted at elderly users. Based on the results of this study, two special functions, namely scene prompting and scene presetting, are added to the smart home system design, which is not usually found in smart home interactive systems. However, these two special functions can significantly improve the satisfaction of elderly users. With the intelligent scene panel as the interaction center and the integration of multimodal interaction, the smart home system with practical functions, convenient operation, precise information, personalized recommendation, and warm care is designed according to the demand characteristics of the elderly users. The final designed smart home interaction system for the age-friendly scenario has such functions as highlighting daily information, pushing reminders by scenes, switching on and off scene modes with one key, environmental monitoring, displaying energy consumption reminders, and body data information. Current research on age-friendly interfaces explores the design of smart home systems for the elderly in terms of their needs and physiological characteristics of life. In 2011, Mayer et al. promoted interoperability and the widespread use of ambient assisted living (AAL) technology by providing an intuitive interface to increase market acceptance [24]. In 2016, Byrne et al. explored whether design principles around AAL systems could be applied to the design of user interfaces for the elderly [25]. In 2017, Gavril et al. proposed a multimodal interface for ambient intelligence and ambient assisted living environments using hand tracking and voice interaction [26]. In 2014, Blasco et al. presented a new design, implementation and evaluation of a smart kitchen that provides ambient assisted living services; an intelligent environment that increases the autonomy of older people and people with disabilities in kitchen-related activities through contextual and user awareness, appropriate user interaction and artificial intelligence [27]. In 2014, McNaull et al. introduced a multi-agent system that provides help and support to the person during the day through the ambient assisted living flexible interface (AALFI), while supplementing the nighttime help provided by NOCTURNAL with feedback help [28]. In order to make the benefits of technological development also benefit the elderly users, and in order to ensure that the elderly have a good interaction performance and experience in the process of using smart home, this study is conducted from both functional and visual perspectives to ensure that the function meets the needs of the elderly and solves their pain points. Then the interface’s visual design meets the operation habits and subjective preferences of the elderly. This paper provides a new idea for the improvement of the user experience of an age-friendly smart home and can guide the design of a complete age-friendly smart home experience closed loop.

## 2. Methods

### 2.1. Experimental Design

Data information in the interactive interface includes information-related numbers, text, graphic elements; the size and arrangement layout of text and graphics, the shape and color of graphics and other presentation methods are all associated elements that affect the cognitive performance of users [29,30]. Sliding is one of the basic operation methods in the interactive interface of the smart home terminal, and the layout of functional modules usually has a vertical and horizontal layout, which usually requires the operation of left and right sliding or up and down sliding to switch pages or find more functional modules [31], so the first part of the experiment is an experimental study designed for the sliding operation method. Function buttons are essential elements in the interactive interface of smart home terminal, and function buttons are the basis used to realize functions when users interact with the smart home terminal interface. Regarding the style of function buttons [17,32], the influence of the different styles of icons on users’ visual search in the touch screen interface argues that the users’ search performance is higher when they search for flat icons. Flat design vs. traditional design is as follows: the comparative experimental study concluded that users’ interaction performance is lower and cognitive load is higher when searching for flat icons, but their study did not conclude for the older user group. Regarding functional button size, while larger sizes may be better for interaction, they may make the interface display denser and lead to a complex interface. Key size is recommended to be larger than 9.5 mm in the American Standard ANSI/HFES 100-2007, and there is no significant improvement in interaction performance when the size is larger than 22 mm. Regarding the key spacing, it is basically concluded in existing studies that there is no significant difference between different key spacing for user interaction performance [33,34].

In order to investigate the effect of different data information on the interaction interface of the age-friendly smart home on the interaction performance of the elderly user group, the experiment investigates the best form of data information that can bring good interaction performance and user preference in terms of sliding mode, button size and button style, to guide and optimize the design to improve the interaction experience of the elderly users using the smart home and reduce the misoperation rate. The metrics used to measure the performance of user information retrieval operations in this phase of the experiment are as follows.

(1) Interactive behavior data

User interaction behavior data mainly include decision speed, click behavior and decision quality. Decision speed refers to the time taken by users to complete the task. The shorter the time, the higher the retrieval interaction performance of users. Click behavior refers to the number of clicks a user makes before completing a task. The fewer the clicks, the higher the user’s retrieval interaction performance. Decision quality is measured by the decision speed and the accuracy rate of task completion. Decision quality is the ratio of accuracy rate to decision speed. The larger the value, the higher the user’s decision quality and the higher the retrieval interaction performance.

(2) Eye-movement index

(a) Saccade is the rapid movement of the gaze point, in which the user usually sweeps from one point of the interface to another, and it is difficult for the user to obtain more information during the eye-hopping process [35,36].

(b) Fixation is a process in which the user’s eyes stay for at least 200 ms, during which the user can obtain a lot of information, which reflects the readability of the observed object [37,38]. The gaze time, the number of gaze points, sweeping paths and gaze hotspot maps were specifically analyzed in the experimental data analysis. Gaze time is the time that subjects look at the area of interest on the experimental interface, which indicates the length of time that subjects spend processing information in the area of interest. The smaller the proportion of gaze time in the experimental data analysis, the less attractive the area is to subjects, and the more the user’s attention is scattered outside the area of interest. Gaze points are used to analyze the depth of information processing in the area, and the higher the proportion of gazes, the better. The simpler the sweep path and the fewer the return paths, the more efficient the subjects’ information acquisition and the better the interaction performance. The hotspot map is used to observe whether the hotspots are concentrated in the target area, and the more the hotspots are concentrated near the area of the icon to be retrieved, the higher the user retrieval performance.

The experiments are divided into two phases. The independent variables in phase 1 are two different layouts and operation styles (sliding up and down and sliding left and right) commonly used in the age-friendly smart home interface. The independent variables are the key size and key style. The functional buttons are square, and the side lengths are set to 10 mm, 12 mm, 15 mm, 18 mm and 22 mm. Four styles of functional buttons are selected. The study is divided into three experiments. Experiment 1 is the effect of swipe style on the interaction performance of the elderly, which is analyzed by decision speed, user click behavior and subjective preference. Experiment 2 is the effect of different button styles on the interaction performance of the elderly, which is analyzed by decision speed, decision quality, number of eye jumps, gaze duration, number of gaze points, user gaze point trajectory and eye movement hot spot map. Experiment 3 is the effect of different button sizes on interaction performance of the elderly, analyzed by decision speed, decision quality, number of eye jumps, gaze duration, number of gaze points, gaze point trajectory and eye movement hot spot diagram. Dependent variables (indicators) were divided into two parts: one was behavioral data to measure the efficiency of user information retrieval and the other was eye-movement data to reflect user interaction performance. The behavioral data include the time spent by subjects to complete the task and the correct rate; the eye-movement data are the hot zones of gaze points and the number of gaze points, the trajectory of gaze point sequences, the number of eye jumps, the gaze rate of interest zones and the average gaze time [39]. The other part is a questionnaire study to collect users’ subjective affective preferences. The interaction behavior data and eye-movement data were collected simultaneously through the ErgoLAB human–computer environment test cloud platform with mobile terminal device usability test eye-movement tracking module. The purpose of the experiment is to match the smart home interaction interface with the age-friendly scenario.

### 2.2. Participants

A total of 17 middle-aged and elderly subjects, aged 53 to 76 years, were recruited for this experiment; 9 of them were male and 8 were female. To ensure the sampling rate and validity of the data, the subjects were required to have no eye swelling or drooping eyelids and no eye surgery, and those with myopia or presbyopia were allowed to wear glasses with natural or corrected visual acuity greater than 1.0; in addition, the subjects were required to have no physical or cognitive impairment and to be right-handed. All subjects were current or retired university faculty and staff or residents of the university. The age range of male subjects was 54 to 72 years, with a mean age of 59.33 and a standard deviation of 6.22. The age range of female subjects was 53 to 76 years, with a mean age of 63.25 and a standard deviation of 8.51. There was no variability in the age of the sample pairs by gender (F = 1.192, *p* = 0.292). The information on the age of the subjects by gender and the analysis of variance by gender are tabulated in Table 1 and Table 2.

### 2.3. Materials and Equipment

**Figure 1:**
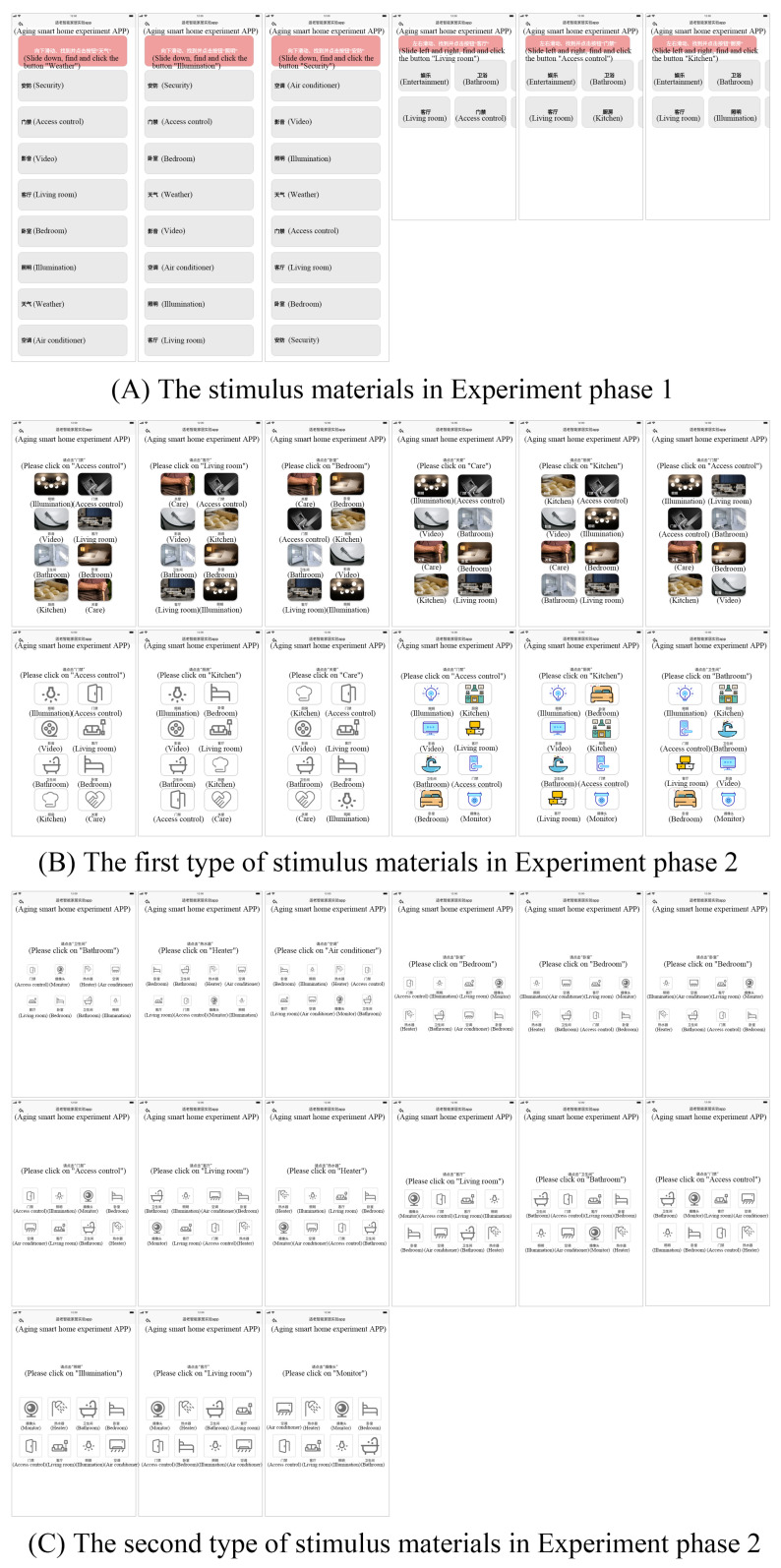
Experimental stage 1 stimulus material.

Experimental stimulus materials: The interface used in the experiment was produced by the ink knife prototype tool and presented on the Honor V6 tablet with a device screen size of 10.4 in and a resolution of 2000 * 1200. The stimulus materials for the first phase of the experiment were two different interface layout forms, three up and down sliding layout interfaces and three left and right sliding layout interfaces, with a total of six test interfaces, as shown in Figure 1A.

The stimulus materials in Experiment phase 2 were interactive interfaces with functional buttons. Each interface of the first type of interface contained 8 functional buttons of the same size and style, and a total of four different button icon styles were set, black letter realistic icon, white letter realistic icon, single color linear icon and colorful faceted icon, with 3 interfaces of each style and a total of 12 interfaces, as illustrated in Figure 1B. Each function button contains icons and corresponding text, and the commonly used functions in smart home are adopted as the contents of the text and icons. Each interface in the second type of interface contains 8 function buttons of the same size, there are 5 side length sizes of 10 mm, 12 mm, 15 mm, 18 mm and 22 mm, and 3 interfaces correspond to each side size function button, totaling 15 interfaces, as shown in Figure 1C.

Experimental equipment: The experimental data acquisition equipment was provided by Beijing Jinfa Technology Co., Ltd., (Beijing, China) and Beijing Institute of Human Factors Engineering and Technology (Beijing, China), and the experimental equipment was Ergo LAB human–machine environment synchronization cloud platform, mobile terminal device usability test eye-tracking module (including mobile terminal test stand and Tobii mobile device test eye-tracking instrument). The other devices were Lenovo Saver y7000 laptop and Honor V6 tablet. The laptop was used as the hardware device for project management, experimental program design, subject information registration, experimental behavior data, eye-movement data and interactive interface data acquisition, signal processing and recording by ErgoLAB cloud platform, and the Honor V6 tablet was the device used for stimulus material presentation for the experiment.

### 2.4. Experiment Process

The experimental tasks that the subjects needed to complete were divided into two phases. In stage 1, the sliding and retrieval test is performed. As shown in Figure 1A, there are several function button modules with text labeling in a vertical layout, and the top of the interface is labeled with specific tasks; subjects need to swipe up and down, find the specified function button and perform single-click operations according to the experimenter’s prompt or the text prompt at the top of the interface. As shown in Figure 1A, there are several function button modules with text labeling in horizontal layout; the subject needs to swipe left and right, find the specified function button and perform a single-click operation according to the experimenter’s prompt or the text prompt at the top of the interface. There are three test interfaces for swiping up and down and swiping left and right; the user needs to complete six swiping and retrieval tests, and the subject needs to complete the task correctly before proceeding to the next task until the end of the six test tasks.

In stage 2, the retrieval and click test is performed. As shown in Figure 1B, there are 12 experimental interfaces; each interface has 8 icons of the same style with text labeling, 3 of each style, and subjects need to retrieve the specified icon and double-click it among the 8 icons according to the experimenter’s prompt or the text prompt at the top of the interface. As shown in Figure 1C, there are 15 interfaces, where each interface has 8 function buttons of the same size with text logos and corresponding icons; subjects need to retrieve the specified icon among the 8 icons and double-click it according to the experimenter’s prompt or the text prompt at the top of the interface. In stage 2, if the subjects did not correct the icons that were not required to be clicked by the instructions, they directly proceeded to the next task and recorded the subject’s response time and correct rate.

After the experiment was completed, subjects were required to complete a subjective preference questionnaire, in order to facilitate the comparison of the results obtained by objective assessment methods with subjective evaluations [40]. The first part of the questionnaire was based on a layout that required sliding up and down and a layout that required sliding left and right. The second part of the questionnaire was based on four different styles of icons for the subjective user preference survey.

## 3. Results

### 3.1. Analysis of Experimental Results of Different Sliding Modes Affecting the Interactive Performance of the Elderly

#### 3.1.1. Decision Speed Analysis

The speed of decision-making is reflected by the time required to complete the task, and the former is inversely proportional to the latter. Before one-way ANOVA, use the box plot to observe the overall data distribution and explore whether there are outliers in the data. After deleting outliers, due to the large sample size, the Kolmogorov–Smirnov normality test is used to test whether the quantitative data at this stage have the nature of the normal distribution [41]. The task response time does not show the significance and has the nature of normal distribution [42]. Secondly, using the homogeneous test of variance [43], the response time of the quantitative data task did not show significance, so the analysis of variance was used for data analysis. The analysis of variance was used to study the difference between the two layouts that need to slide up and down and the two layouts that need to slide left and right on the response time of the subjects to complete the function key retrieval and click task. It was found that there was no significant difference in the response time between different operation modes (*p* > 0.05). In addition, through the analysis of variance, the task completion time of the two operation modes has nothing to do with age and gender. Table 3 shows the variance analysis of task completion time in different sliding modes.

Analyze the relationship between task type and task completion time. In phase I of the experiment, the average time for subjects to complete each task is 9.86 s, the overall average time for subjects to complete the up and down sliding tasks is 10.35 s, and the overall average time for subjects to complete the left and right sliding tasks is 9.37 s. According to the duration of completing the task, the time taken by the subjects to complete the up and down sliding task is 0.98 s higher than that to complete the left and right sliding task, which is 0.49 s higher than the average time. Although the former takes a long time, the difference between the two is not obvious.

#### 3.1.2. Click Behavior Analysis

First of all, the number of hits of quantitative data subjects is tested for normality. Because the sample size is large, Kolmogorov–Smirnov normality is used to test whether the data at this stage have the nature of normal distribution [42]. The number of hits of subjects does not show the significance and has the nature of the normal distribution. Afterwards, using the homogeneous test of variance, the response time of the quantitative data task did not show significance, so the analysis of variance was used for data analysis. In phase I of the experiment, the average number of clicks of subjects completing each task is 4.29 times, the overall average number of clicks of subjects completing up and down sliding tasks is 3.57 times, and the overall average number of clicks of subjects completing left and right sliding tasks is 5.02 times. According to the number of clicks required to complete the task, the number of clicks required to complete the up and down sliding task is 1.45 times less than that required to complete the left and right sliding task, the former is significantly less than the latter, and the number of clicks required to complete the up and down sliding task is 0.72 times less than the average number of clicks. The variance analysis was used to study the difference between the two layouts that need to slide up and down and the layout that needs to slide left and right on the number of clicks required by the subjects to complete the function key retrieval and click task. It was found that different operation modes showed significant differences in the number of clicks (*p* < 0.01). To sum up, the two layouts that need to slide up and down and the two layouts that need to slide left and right have little difference in the time spent to complete the task, but there is a large difference in the number of clicks, and the former is better than the latter. Table 4 shows the variance analysis of clicks in different sliding modes.

#### 3.1.3. User Subjective Preference Analysis

A subjective questionnaire survey was conducted on the subjects to investigate the user’s subjective preferences in terms of comfort and usability. There were 2 corresponding questions in terms of comfort and usability, with a score of 1–5 points. It can be seen from the table that the analysis of variance is used to study the differences between the four descriptions of task types: the samples of different task types do not show the significance for “Make me feel beautiful” and “I think the content is readable” (*p* > 0.05), which means that different task types show consistency and no difference in aesthetics and readability. In addition, the task type samples showed significantly (*p* < 0.05) for “Make my eyes feel comfortable” and “I am satisfied with the time spent completing the task”, which means that there are differences in the satisfaction of comfort and time-consuming among different task type samples: the task type showed a significant level of 0.01 for comfort (F = 14.520, *p* = 0.001). The specific comparison difference shows that the average value of the up-down sliding type (5.00), will be significantly higher than the average value of left-right sliding (4.15). Task types showed a significant level of 0.05 for 4 (F = 4.909, *p* = 0.036), and the upper and lower average values (4.85) were significantly higher than the left and right average values (4.38). Table 5 shows for variance analysis of subjective preference of different sliding modes.

According to the comparison of the average values of comfort and availability, as shown in Table 6, the upper and lower sliding types score 4.75 points in comfort and availability, while the left and right sliding types score 4.285 points in comfort and 4.565 points in availability, which are lower than the upper and lower sliding types.

### 3.2. Analysis of Experimental Results of Different Key Styles Affecting the Interactive Performance of the Elderly

#### 3.2.1. Saccade Data Analysis

The saccade is a saccade action performed when both eyes are synchronized, and the subjects’ fixation points move rapidly. First of all, the normality test is carried out on the quantitative data of eye movement experiment of different style samples in the second experimental stage, and the Kolmogorov–Smirnov normality is used to test whether the data in this stage have the nature of the normal distribution [44]. Table 7 shows the kurtosis absolute values of the eye movement data are less than 10 and the skewness absolute values are less than 3, indicating that the data can basically be accepted as normal distribution.

Furthermore, the variance homogeneity is used to test whether there is a significant difference in the data fluctuation of the number of saccades of the subjects. In Table 8, types 1 to 4 are five types of experimental interfaces containing function keys of different sizes, corresponding to four styles respectively, the same below. It can be seen that different types of samples show a significant effect on the number of saccades (*p* < 0.05), that is, the fluctuation of different types of samples for the number of saccades is inconsistent. Therefore, Welch ANOVA is used to analyze the difference in the number of saccades.

Using Welch ANOVA for analysis, as shown in Table 9, the number of saccades of different types of experimental interfaces shows significant (F = 3.575, *p* = 0.002). The comparison results of the average scores of groups with obvious differences are type 2 > type 1; type 2 > type 3; type 4 > type 3.

**Figure 2:**
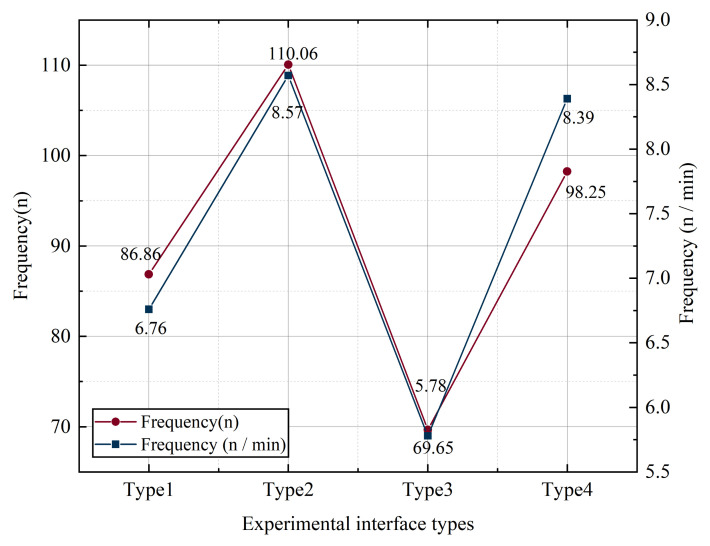
The number and frequency of saccades with different key styles.

Based on the results of the data analysis, a univariate analysis was performed to analyze the relationship between icon style and saccade times. Figure 2 shows the number of saccades and the frequency (n/min) of subjects with different interface types. The lowest number of saccades and the frequency of subjects were observed when the experimental interface was type 3; the highest number of saccades and the frequency of subjects were observed when the experimental interface was type 2.

#### 3.2.2. Fixation Duration Data Analysis

Before statistical data, draw areas of interest (AoI) for each experimental interface. At this stage, the area of interest is the area of the corresponding icon that the subjects need to retrieve. Count the proportion of time the subjects stare at the area of interest during the experiment. For a start, the normality test was carried out on the proportion of the length of fixation in the region of interest. The fixation time was not significant (*p* > 0.05), which means that the fixation time has the characteristics of normality. Table 10 shows the normality test of the key fixation duration data of different styles.

Additionally, variance homogeneity is used to test whether there is a significant difference in the fluctuation of data. It can be seen from Table 11 below that the samples of different styles will not show a significant effect on the AoI fixation time (*p* > 0.05). Therefore, analysis of variance is used to study the difference.

**Figure 3:**
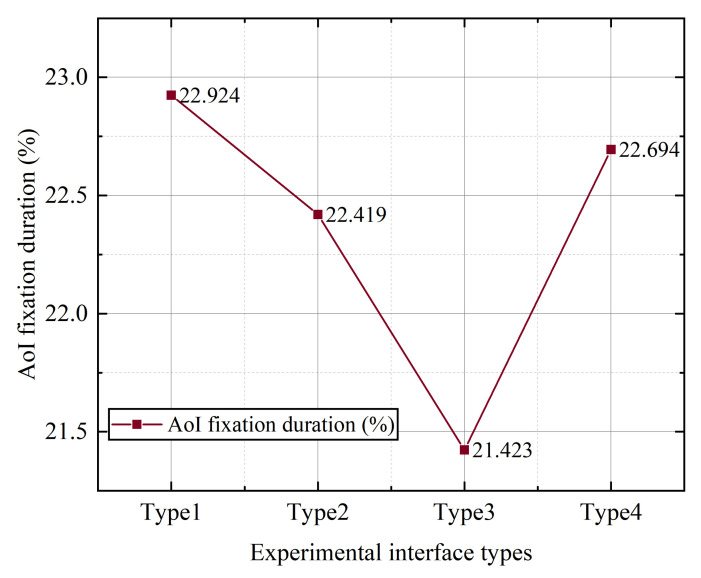
The average value of the ratio of the gaze duration of different styles of buttons.

It can be seen from Table 12 below that by using the difference of one-way ANOVA type for fixation time [45], samples of different wind types show the significant differences for fixation time (F = 4.842, *p* = 0.033), which means that different types of samples have significant difference for fixation time. For visual display by using a broken-line diagram, as shown in Figure 3, it can be seen that the AoI fixation time proportion of the type 3 interface is the lowest, which is significantly lower than that of the other three types of interfaces. The difference between type 1, type 2 and type 4 is not obvious.

#### 3.2.3. Fixation Point Data Analysis

The proportion of fixation points is the ratio of the fixation points of AoI to the number of fixation points of the whole interface. The higher the proportion of fixation points, the higher the efficiency of information processing in this area and the deeper the depth of information processing. Firstly, the normality test was conducted for the proportion of subjects’ viewpoints in the quantitative data. The Shapiro–Wilk normality test was used to test whether the data in this stage had the nature of normal distribution [46]. The fixation point proportion did not show significance (*p* > 0.05), which means that the fixation point proportion has the characteristic of normality. Table 13 shows the normality test of the proportion data of different styles of key fixation points.

The variance homogeneity is used to test whether there are obvious differences in the fluctuation of the subject’s viewpoint proportion data. Types 1 to 4 in the table are four types of experimental interfaces containing different style icons. It can be seen from Table 14 below that the proportion of fixation points of different style samples is significant (*p* < 0.05), which means the data volatility of different style samples for the number of viewpoints of the subjects is inconsistent, so Welch ANOVA was used to analyze the difference.

Table 15 shows that Welch ANOVA analysis of variance was used to study the difference in fixation ratio between styles. It was found that samples of different styles did not show the significant differences in fixation ratio (*p* > 0.05), which means that different types of samples showed consistency in the fixation ratio. Table 15 shows the Welch variance analysis of the proportion of fixation points of keys with different styles.

**Figure 4:**
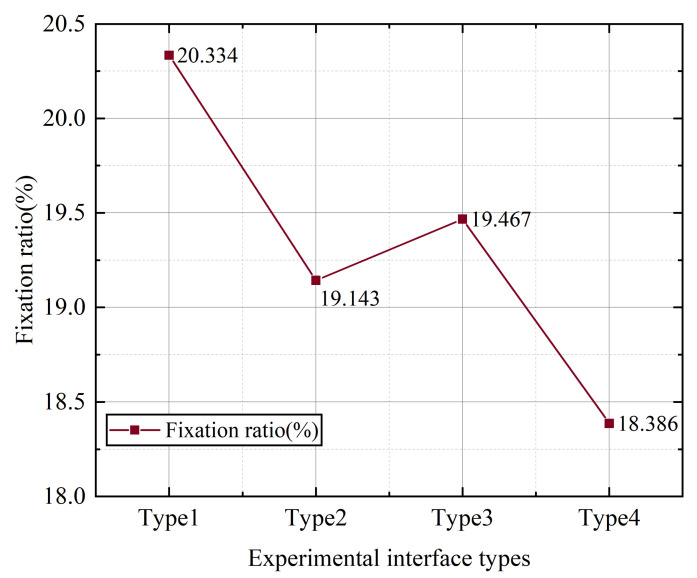
The average value of the gaze point ratio of different styles of buttons.

According to the data analysis results, a univariate analysis was performed to analyze the relationship between icon style and fixation point ratio. Figure 4 shows the fixation ratio of the subjects during the experiment with different styles and types of interfaces. It can be seen that the fixation ratio of style type 4 is the lowest, which is 18.39%; the fixation ratio of type 1 is the highest, which is 20.33%.

**Figure 5:**
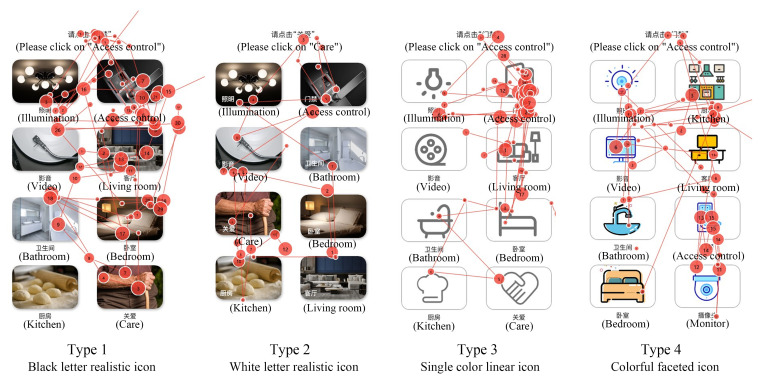
Scanning paths of 4 types of button interfaces.

The saccade path can reflect the temporal and spatial characteristics of eye movement when subjects complete the experiment. As shown in Figure 5, in the scanning paths of the subjects of 12 interfaces in the experiment at this stage, it can be seen that the fixation paths of interface type 1 and type 3 are more circuitous, and the decision-making efficiency of the subjects is low. Type 2 has shorter relative paths, fewer look-back paths and higher information acquisition efficiency. In type 4, the scanning path of subjects in two of the interfaces is better, but the efficiency of obtaining information of subjects in one interface is low. According to the later investigation, the reason is that the subjects do not understand the meaning of the icons in place.

**Figure 6:**
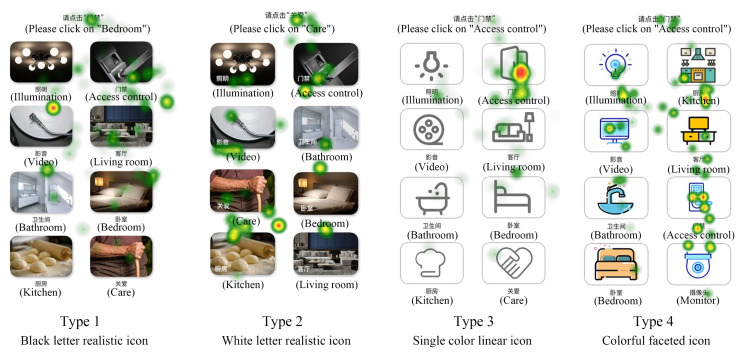
Gaze data heat map of 4 styles of button interface.

As shown in Figure 6, the eye movement hot spots of subjects on 12 interfaces at this stage reflect the characteristics of eye movement space and duration when subjects complete the experiment through cloud identification. Different areas on the picture are marked and presented according to the subjects’ attention and duration. The marking means are color change and color opacity change, as well as the location and density of cloud pattern. The duration is marked by color. Red indicates that the subjects pay attention to a point for the longest time, followed by yellow and green again. The hot spot map was used to judge whether the subjects focused on the icons to be retrieved in the experiment. It can be seen from the pictures that the subjects perform best in the type 3 interface, and their attention is basically focused on the icons they need to find. The rest of the areas have a short sight stay time, the highest accuracy of information search and high efficiency of information search. The subjects performed well in the type 4 interface. In two interfaces, the subjects basically focused on the icons they needed to find, and in the other interface, the subjects focused on several different icons. The subjects’ attention performance is also good in the type 2 interface. The subjects’ eyes of the 3 interfaces are concentrated in or near the icon area to be retrieved, but the subjects’ eyes of one interface are relatively scattered. The search efficiency of the subjects in the type 1 interface is the lowest, the line of sight is scattered, and the viewpoint is concentrated in the area of non-target icons. In conclusion, the subjects performed the best eye movement on the type 3 interface.

#### 3.2.4. User Subjective Preference Analysis

A subjective questionnaire survey was conducted on the subjects to investigate the subjective preferences of users in terms of comfort, usability and overall evaluation. The comfort corresponds to questions 1 to 2, “Make my eyes comfortable” and “Make me feel beautiful”, with a score of 1–5; usability corresponds to questions 3 to 8, “I think the content is readable”, “I am satisfied with the time spent completing the task”, “I am satisfied with the auxiliary retrieval function of the picture”, “This style makes me complete the task more effectively”, “This style makes it easier for me to complete the task”, and “This style makes it easy for me to find the task information”, with a score of 1–5; The overall evaluation corresponds to the last question, “I’m very satisfied with this style”, with a score of 1–5. First, test the normality to see whether the data have the nature of the normal distribution. The absolute values of kurtosis of the data in questions 1–9 are less than 10 and the absolute values of skewness are less than 3, so the data can basically be accepted as the normal distribution. The results are shown in Table 16.

Further, variance homogeneity is used to test whether there are significant differences in the fluctuation of the data. From the table, it can be seen that the samples of different styles will not show significance (*p* > 0.05) for the 5 items of item 1, item 2, item 3, item 4, and item 9. The variance is homogeneous, so the analysis of variance is used to study the difference. Different style samples showed significant (*p* < 0.05) for 4 items, item 5, item 6, item 7 and item 8; this means that the data volatility of different style samples for 4 items, item 5, item 6, item 7 and item 8, is inconsistent. Welch ANOVA was used to study the difference relationship. The results are shown in Table 17.

It can be seen from Table 18 that using one-way ANOVA to study the differences of 5 data items, item 1, item 2, item 3, item 4 and item 9, different style samples will not show the significant differences for all the above 5 items (*p* > 0.05), which means that there is no significant difference between different style samples for item 1, item 2, item 3, item 4 and item 9.

It can be seen from Table 19 below that using Welch ANOVA to study the differences of 4 items, item 5, item 6, item 7, and item 8, it is found that the samples of different styles will not show the significant differences for item 6, item 7, and item 8 (*p* > 0.05), which means that the samples of different styles show consistency and no significant difference for item 5, item 6, item 7 and item 8. However, different style samples show the significance for topic 5 (F = 3.617, *p* = 0.029), which means different style samples have obvious differences for topic 5. Therefore, for the auxiliary role of pictures, the score of type 1 is significantly higher than that of type 4. Subjects think that type 3 and type 4 play a better role in picture assistance.

**Figure 7:**
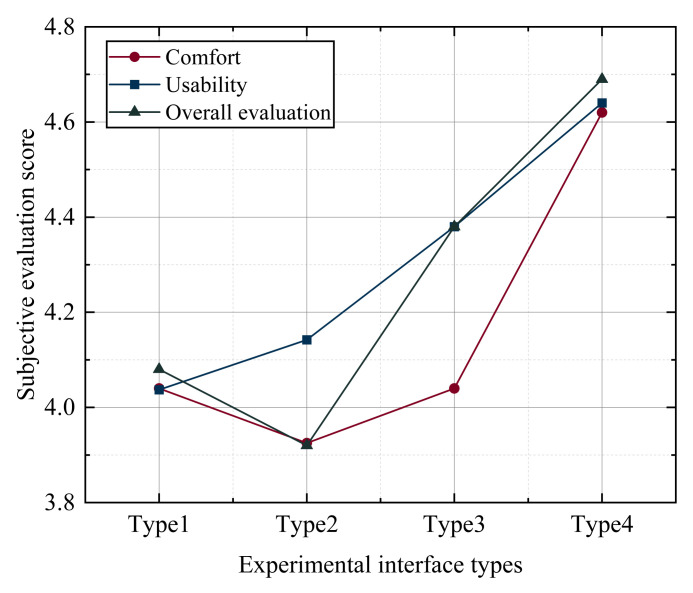
Subjective evaluation of different styles of keys.

Analyze the comfort, usability and overall evaluation of the 4 style types of interfaces, and the scores are shown in Table 20. As shown in Figure 7, type 2 has the lowest scores in terms of comfort and overall evaluation, while type 4 has the highest scores in terms of comfort, availability and overall evaluation. On the whole, type 1 and type 2 have low scores in terms of users’ subjective preferences.

### 3.3. Analysis of Experimental Results of Different Key Sizes Affecting the Interactive Performance of the Elderly

#### 3.3.1. Decision Speed Analysis

The speed of decision-making is inversely proportional to the time required to complete the task. Due to the large sample size, Kolmogorov–Smirnov normality is used to test whether the quantitative data in this stage have the nature of normal distribution [30]. The task response time is not significant and has the nature of the normal distribution. Secondly, using the homogeneous test of variance, the response time of the quantitative data task did not show significance, so the analysis of variance was used for data analysis. In the experiment, the subjects completed the type 1 icon retrieval task with an average time of 7.94 s, the longest time; the overall average time to complete the type 4 icon retrieval task is 4.65 s, which is the shortest. The analysis of variance was used to study the relationship between the different size types and the response time of the subjects to complete the function key retrieval and click task. It was found that there was no significant difference in the response time between different operation modes (*p* > 0.05). Table 21 shows the rectangular difference analysis when making decisions with different key sizes.

#### 3.3.2. Decision Quality Analysis

**Figure 8:**
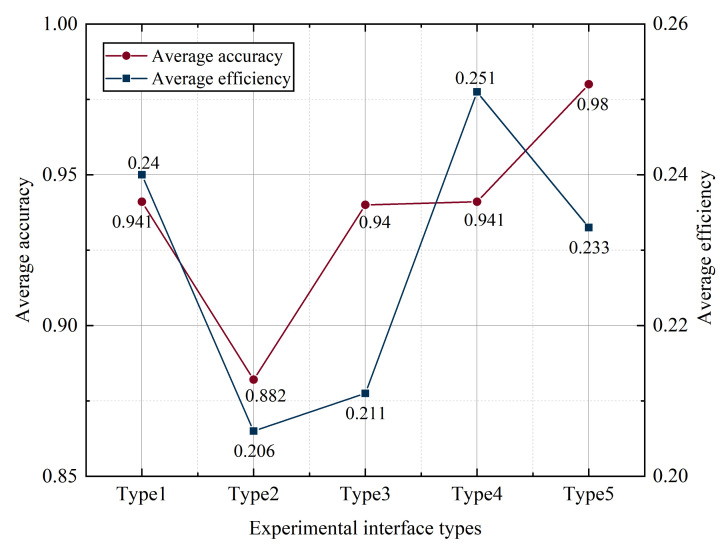
Decision quality of buttons of different sizes.

The accuracy and efficiency of the tasks performed by the subjects are used to assess their decision-making abilities. The decision-making efficiency is the accuracy of the tasks completed by the subjects divided by the time taken by the subjects to complete the tasks. As shown in Figure 8, through single-factor analysis, it can be seen that the size of the icon does not show a linear relationship with the task completion accuracy and decision-making efficiency. When the experimental interface is type 5, the accuracy is the highest, but the decision-making efficiency is only the third among all types; when the experimental interface is type 4, the accuracy and decision-making efficiency are higher; and when the experimental interface is type 2, the average correctness and decision efficiency are the lowest.

#### 3.3.3. Saccade Frequency Data Analysis

Primarily, the normality test is carried out on the quantitative data of eye movement experiments of different sizes of samples in the second experimental stage. Because the sample size is large, the Kolmogorov–Smirnov normality test is used to test whether the data in this stage (statistical product service solutions (SPSS) references) have the nature of normal distribution [31]. The p values of the two kinds of data of eye movement experiment and eye jump frequency are less than 0.01, but it is difficult to meet the requirements of the normality test; the absolute values of kurtosis of the two types of data are less than 10 and the absolute values of skewness are less than 3, indicating that although the data are not an absolute normal distribution, they can basically be accepted as a normal distribution. Table 22 shows the normality test of eye jump data of keys with different sizes.

Additionally, the variance homogeneity is used to test whether there is a significant difference in the fluctuation (standard deviation) of the data of eye hop times and eye hop times. Types 1 to 5 in the table are five types of experimental interfaces containing function keys of different sizes, corresponding to the side length of 10 mm, 12 mm, 15 mm, 18 mm and 22 mm respectively, the same below. As can be seen from the following table: samples of different sizes do not show significant effect on the number of saccades (*p* > 0.05), which means that the fluctuation of the number of saccades of different types of sample data is consistent. The variance of the number of saccades of subjects under samples of different sizes is uniform, so it meets the prerequisite requirements of analysis of variance, so analysis of variance is used to study the difference. Different size samples showed significant (*p* < 0.05) for the frequency hopping times (n/min), that is, the data volatility of different types of samples for the frequency hopping times (n/min) of the tested eyes was inconsistent. Consequently, Welch ANOVA was used to analyze the difference in frequency hopping times of the tested eyes. The results are shown in Table 23.

To sum up: the number of saccades basically meets the normality, and the samples of different sizes have the uniformity of variance for the number of saccades, which meets the premise requirements of analysis of variance; the subjects’ eye frequency hopping times basically meet the normality, but the samples of different sizes do not have the uniformity of variance for the subjects’ eye frequency hopping times, and the data fluctuation is obviously inconsistent. Therefore, Welch ANOVA or nonparametric test are used to study the difference relationship.

Using one-way ANOVA, as shown in Table 24, the number of saccades of different sizes and types of experimental interfaces is not significant (*p* > 0.05), which means that the number of saccades of different sizes and types of interfaces is consistent and there is no difference. As shown in Table 25, the Welch ANOVA study shows that interfaces of different sizes and types will not show a significant effect on frequency (n/min). To sum up, different types of samples show consistency in frequency and frequency (n/min), and there is no difference. It shows that different sizes will not affect the saccade action of subjects in the process of completing the task.

**Figure 9:**
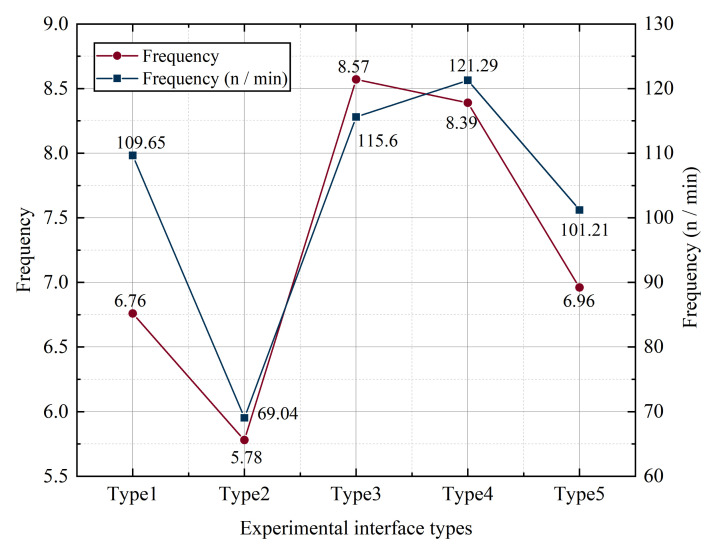
Mean value of eye movement data for different button sizes.

According to the data analysis results, single-factor analysis was carried out to analyze the relationship between icon size and saccade frequency and saccade frequency. Figure 9 shows the average value of the number of eye jumps and the frequency of eye jumps in eye movement data. It can be seen that the type 2 interface has the lowest average number of eye jumps and the lowest frequency of eye jumps. The type 3 interface has the most eye jumps, 8.65, which is close to twice the number of eye jumps of type 2. The type 4 interface has the highest saccade frequency.

#### 3.3.4. Fixation Duration Data Analysis

Before the statistics, draw AoI for each experimental interface. The area of interest at this stage is the area of the corresponding icon that the subjects need to retrieve. Count the proportion of time the subjects stare at the area of interest during the experiment. Firstly, the normality test was carried out on the proportion of the length of fixation in the region of interest. The fixation time was not significant (*p* > 0.05), which means that the fixation time has the characteristics of normality. The results are shown in Table 26.

Subsequently, variance homogeneity is used to test whether there is a significant difference in the fluctuation of data. It can be seen from Table 27 below that samples of different sizes will not show a significant effect on AoI fixation time (*p* > 0.05). Therefore, analysis of variance is used to study the difference. It can be seen from Table 28 that there is no significant difference in fixation time for different types of samples.

**Figure 10:**
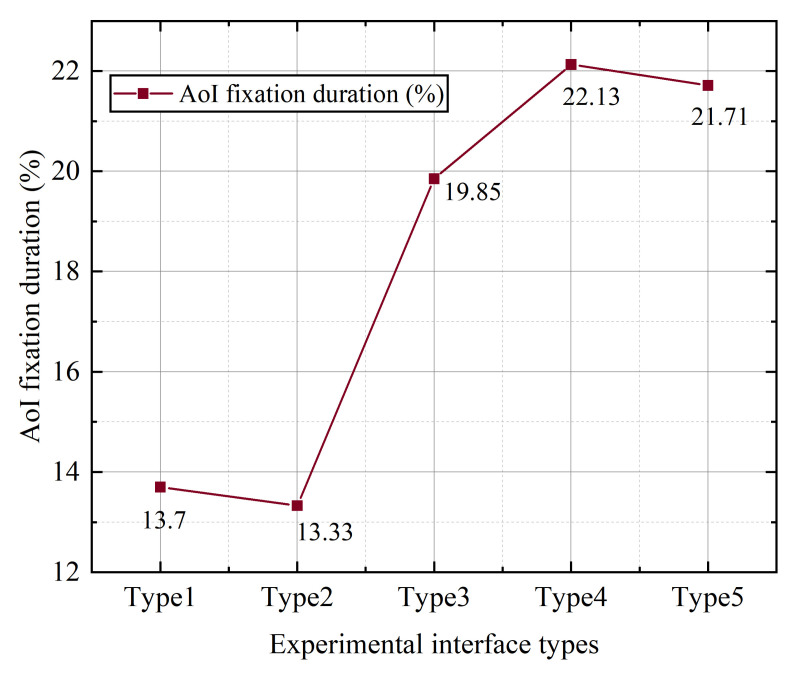
The average value of the ratio of the gaze duration of the buttons of different sizes.

According to the data analysis results, a single factor analysis was carried out to analyze the relationship between icon size and AoI fixation duration ratio. As shown in Figure 10, the proportion of subjects’ fixation time on AoI in the duration of interface experiments with different sizes and types is shown. It can be seen that the proportion of fixation time on AoI increases with the increase in icon size, but the fixation time of type 2 is shorter than that of type 1, and that of type 5 is shorter than that of type 4.

#### 3.3.5. Viewpoint Data Analysis

At the outset, the normality test is carried out for the proportion of points of view of quantitative data. Since the sample size is less than 50, the Shapiro–Wilk normality test is used to test whether the data at this stage have the nature of normal distribution [34]. The p-value of the number of points of view of quantitative data is less than 0.05, the absolute value of kurtosis is is less than 10 and the absolute value of skewness is less than 3, indicating that although the data are not an absolutely normal distribution, they can basically be accepted as the normal distribution. The results are shown in Table 29.

Afterward, the variance homogeneity is used to test whether there is a significant difference in the fluctuation (standard deviation) of the subject’s viewpoint proportion data. Size 1 to size 5 in the table are 5 types of experimental interfaces containing icons of different sizes. As can be seen from Table 30 below: different types of samples will not show a significant effect on the number of fixation points (*p* > 0.05), which means that the volatility of data of different types of samples shows consistency, no difference and homogeneity of variance. To sum up, the data meet the premise requirements of using analysis of variance.

It can be seen from Table 31 below that the analysis of variance is used to study the difference between the size and the proportion of fixation points. It is found that the proportion of fixation points for samples of different sizes will not be significant (*p* > 0.05), which means that the proportion of fixation points for different types of samples is consistent and there is no difference.

**Figure 11:**
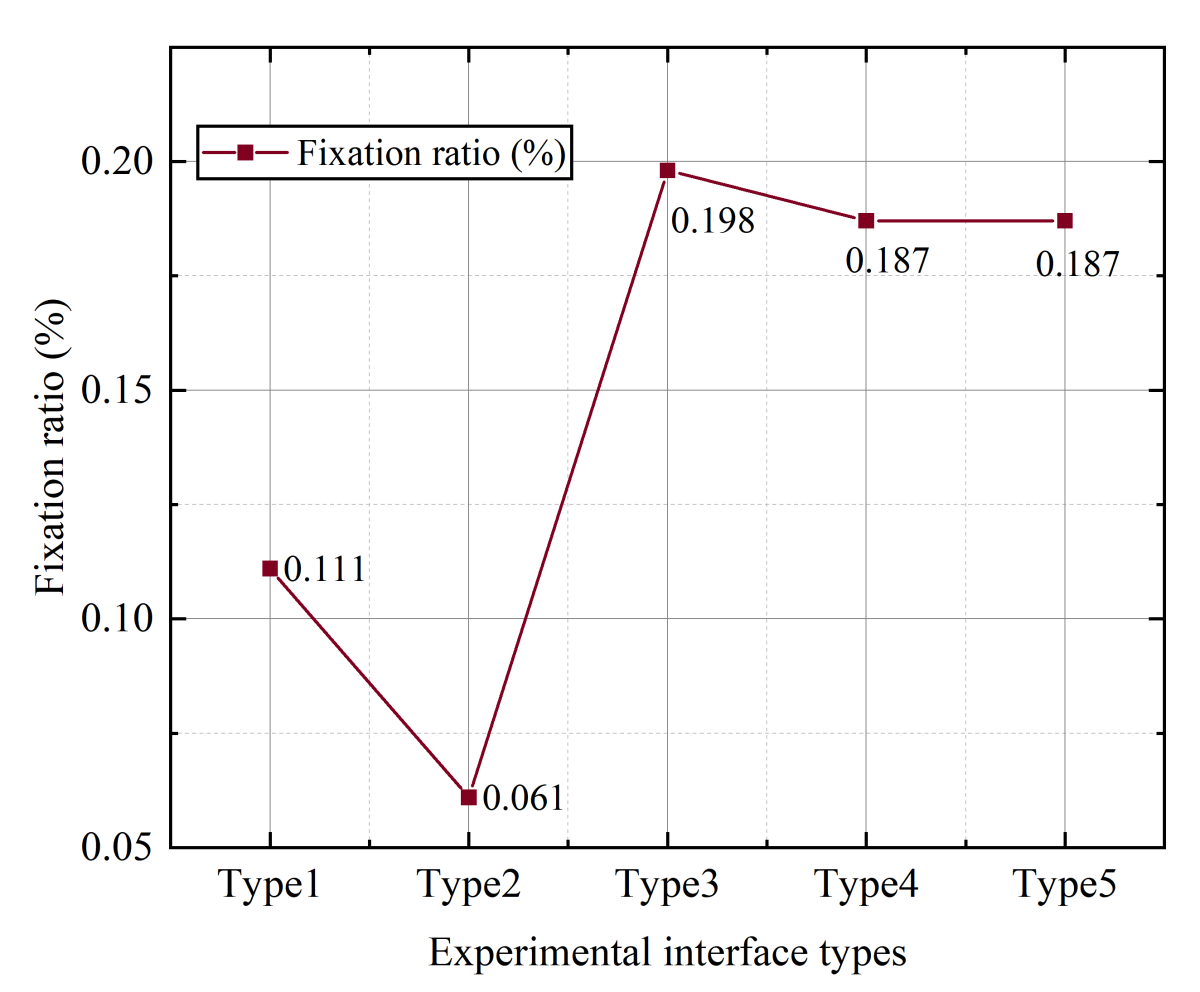
The average value of the fixation point ratio of buttons of different sizes.

According to the results of the data analysis, a single factor analysis was conducted to analyze the relationship between the icon style and the proportion of attention points as shown in Figure 11, which shows the average proportion of attention points of the subjects during the experiment of different size types of interfaces. It can be seen that size type 2 has the lowest proportion of gaze points and type 3 has the highest proportion of gaze points.

**Figure 12:**
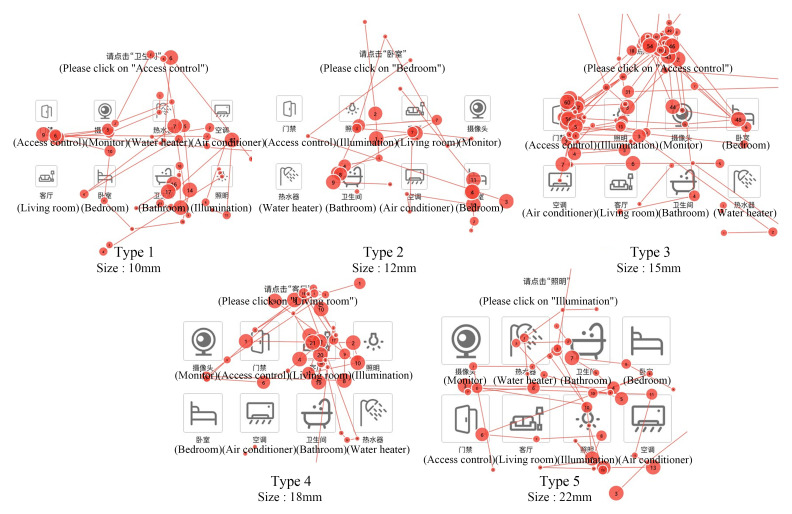
Eye movement saccade paths in the experimental interface of buttons of different sizes.

The saccade path can reflect the temporal and spatial characteristics of eye movement when subjects complete the experiment. As shown in Figure 12, in the scanning paths of the subjects of 15 interfaces in the experiment at this stage, it can be seen that the path of interface type 2 is shorter, there are fewer looking-back paths, and the information acquisition efficiency is higher. Although the key size of type 3, type 4 and type 5 is larger, the number of fixation points is more than that of type 2, and the fixation path is more circuitous, which is not conducive to users’ decision making. Although the key size of type 2 interface is not the largest, in contrast, the scanning path is better, the number of fixation points is less, and the decision-making efficiency is higher.

**Figure 13:**
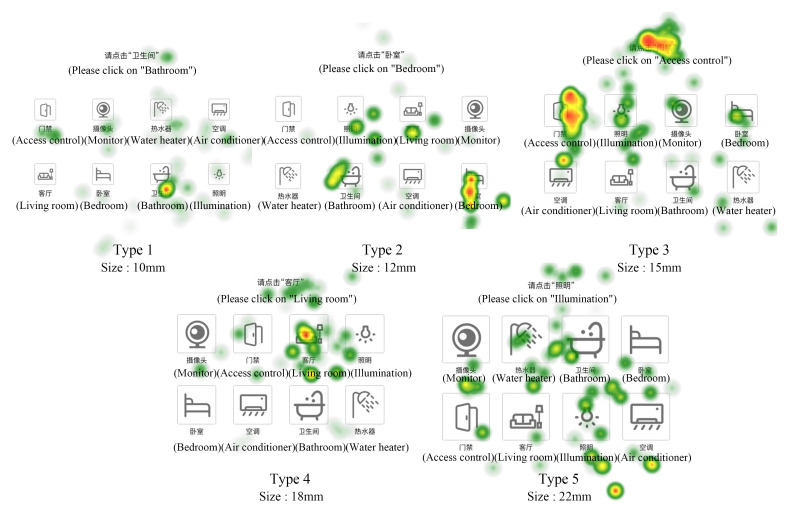
Eye movement heat map of different size button experiment interface.

The hot spot map reflects the characteristics of eye movement space and time length when subjects complete the experiment through cloud identification. As shown in Figure 13, the eye-movement characteristics of the subjects on the 15 interfaces at this stage are marked and presented according to the subjects’ attention and duration. The marking means are color change and color opacity change, as well as the position and density of the cloud pattern. The duration is marked by color. Red indicates that the subjects pay attention to a point for the longest time, followed by yellow and green again. The hot spot map is used to observe whether the user’s attention is focused on the icon to be retrieved. It can be seen from the picture that the subjects’ attention performance in the type 1 interface is general. In only one interface, the subjects focus on the icon they need to find, and in the other two interfaces, their attention is distracted or even focused on other icons. In the type 2 interface, the tested users performed well and basically focused on the icons they needed to find. In the type 3 interface, subjects only focus on the icon they need to find on one of the interfaces, while for the attention on the two interfaces, one focuses on several different icons and the other focuses on non-target icons, with general search accuracy. On the experimental interface of type 5, the fixation points of the subjects are very scattered and do not obviously focus near the target icon, indicating that the search efficiency of the subjects on this type of interface is low, which also shows that the larger the icon, the better. To sum up, the subjects performed the best eye movement on the type 2 interface, with the highest accuracy and efficiency of information search.

## 4. Conclusions

To solve the pain points of middle-aged and elderly users when using smart home products and interactive systems, reduce the impact of the “digital divide”, enjoy the benefits of technological development and create a comfortable and healthy smart living solution for them, the design of the smart home interaction interface should follow the design ideas of functionally solving the problems described in the objective scenarios and improving user interaction performance in the interface interaction. The principles of smart home interaction design based on age-friendly scenarios are that the overall design should be reasonable, fitting the characteristics of the target users and referring to the user model; the interaction operation should be efficient, reducing unnecessary interaction steps, making the design efficient, convenient and simple to operate; the interaction details should be unified, reducing user learning costs; the interface should be visually simple and intuitive with clear information; and in terms of personalized needs, it can be customized to meet different user. In this paper, we summarize three interface factors that are better for the age-friendly scenario based on the results of experiments and data analysis. In terms of sliding style, the up-and-down sliding layout interface has better interaction performance and subjective evaluation than the left and right sliding layout interface, so the smart home interface design should give priority to vertical layout and up and down sliding operation method; in terms of button style, line type simple button style has the best interaction performance and subjective evaluation, so the icon design should favor simple line style; in terms of button size, the interaction performance of function buttons is 12–18 mm. Therefore, the icon size should be 12 mm to 18 mm for the prototype design.

The design of the smart home interaction interface needs to follow the design ideas of functionally solving the problems described in the objective scenario and improving user interaction performance in the interface interaction. The smart home interaction design principles based on age-appropriate scenarios are as follows: overall reasonable design, fitting the target user characteristics and referring to the user model; efficient in interaction operation, reducing unnecessary interaction steps, making the design efficient, convenient and simple to operate; unified in interaction details, reducing user learning costs; simple and intuitive in interface visualization, with clear information; in terms of personalized needs, customizable to meet different users.

Originally, for different sliding layout methods, the objective data and subjective preference results are basically the same. The difference between the different sliding operation methods in subjects’ task completion time was not significant, but there was a significant difference in the number of clicks required before subjects completed the task (F = 20.22, *p* = 0.00), and the number of clicks for the up-down sliding operation and layout method (n = 3.57) was significantly less than that for the left-right sliding (n = 5.02), which means the user’s retrieval interaction in the up-down sliding operation method performance is higher. In terms of subjective preference, there is a significant difference between different sliding types for comfort, with the score of up and down sliding (5.00 points) being significantly higher than that of left and right sliding (4.15 points); there is a significant difference in time consumption satisfaction, with the score of up and down sliding (4.85 points) being significantly higher than that of left and right sliding (4.38 points), and the scores of both up and down sliding are higher than those of left and right sliding in terms of comfort and usability evaluation. In terms of comfort and usability, the scores of up and down sliding are also higher than those of left and right sliding. Therefore, the interaction performance and subjective evaluation of the up-down sliding layout are better.

Moreover, for different function button styles, there was a significant difference in the number of eye bounces by style type (F = 3.58, *p* = 0.002); the number of eye bounces was the lowest for the style type of monochrome lines (n = 5.78), and the number of eye bounces was the highest for the style type of dark physical pictures with light text (n = 8.57). In terms of scanning paths, the function buttons with dark physical images and light text performed better; the function buttons with monochromatic lines performed best in the hot spot analysis. In terms of subjective user preference, subjects thought that type 3 (function buttons with monochromatic lines) and type 4 (function buttons with colored lines) had good picture-assisted retrieval, and in terms of overall satisfaction, type 4 (function buttons with colored lines) scored the highest. The scores of type 1 (physical picture with dark text) and type 2 (physical picture with light text) were lower. During the subjective questionnaire, many subjects actively expressed that they disliked the style of type 2 the most and did not like dark images, preferring a brighter style. In addition, male subjects preferred monochromatic line icons and female subjects preferred colorful icons. In summary, the interaction performance and subjective evaluation of the line type minimalist button style were the best.

For different function key sizes, it was found that the subjects need the longest time (7.94 s) to complete the icon retrieval task of the interface with size type 1, which means a function key size of 10 mm, and the shortest time (4.65 s) for type 4, which means a function key size of 18 mm. As for the quality of decision-making, type 4 has high accuracy (0.94) and decision-making efficiency (0.25). There is no significant difference in the number of saccades among different size types; 18 mm interface is the highest proportion of AoI fixation duration (22.31%). The proportion of fixation points at 15 mm interface is the highest. In terms of scanning path, although the key size of 12 mm interface is not the largest, the scanning path is the best; through the hot spot map, it is found that the subjects with a 12 mm interface have the best eye movement performance. In conclusion, the interaction performance is better when the function key is 12–18 mm.

For the different functional button sizes, it was found that subjects took the longest time (7.94 s) to complete the icon retrieval task for size type 1, which means the interface with a functional button size of 10 mm, and the shortest time (4.65 s) for size type 4, which means the interface with a functional button size of 18 mm. Regarding the decision quality, both the correct rate (0.94) and the decision efficiency (0.25) were higher for type 4. There was no significant difference in the number of eye jumps among different size types. The 18 mm interface had the highest proportion of AoI gaze duration (22.31%) and the 15 mm interface had the highest proportion of gaze points. In terms of sweeping path, the 12 mm interface had the best sweeping path although the key size was not the largest. The 12 mm interface had the best eye movement performance as found by the hot spot map. In summary, the interaction performance is better when the function keys are 12–18 mm.

From the perspective of smart home interactive interface function realization, the conclusion of this experiment about the better interface data information improves the rationality of the age-friendly smart home interface and makes the smart home interface better cope with the age-friendly scenario.

## Figures and Tables

**Table 1:** Subject sex and age information table.

Number	Subjects	Gender	Age/Years	Number	Subjects	Gender	Age/Years
01	Mr. Zhang	Male	54	10	Ms. Zhu	Female	53
02	Mr. Min	Male	55	11	Ms. Law	Female	57
03	Mr. Gu	Male	55	12	Ms. Tong	Female	57
04	Mr. Qiu	Male	56	13	Ms. Peng	Female	60
05	Mr. Zhang	Male	57	14	Ms. Liu	Female	60
06	Mr. Li	Male	57	15	Ms. Yang	Female	70
07	Mr. Hu	Male	61	16	Ms. Yang	Female	73
08	Mr. Wang	Male	67	17	Ms. Liu	Female	76
09	Mr. Hu	Male	72				

**Table 2:** Gender analysis of variance.

	Gender (Mean ± SD)		F	*p*
	Female	Male		
Age (year)	63.25 ± 8.51	59.33 ± 6.22	1.192	0.292

*p* < 0.05, *p* < 0.01.

**Table 3:** Variance analysis of task completion time in different sliding modes.

	Task Type (Mean ± SD)		F	*p*
	Sliding Up and Down	Sliding Left and Right		
Task completion time (s)	10.35 ± 11.14	9.37 ± 4.19	0.332	0.56

*p* < 0.05, *p* < 0.01.

**Table 4:** Analysis of variance for clicks with different sliding methods.

	Task Type (Mean ± SD)		F	*p*
	Sliding Up and Down	Sliding Left and Right		
Number of hits (n)	3.57 ± 1.49	5.02 ± 1.76	20.222	0.000 **

** *p* < 0.01.

**Table 5:** ANOVA of subjective preference in different sliding modes.

	Task Type (Mean ± SD)		F	*p*
	Sliding Up and Down	Sliding Left and Right		
Make my eyes feel comfortable	5.00 ± 0.00	4.15 ± 0.80	14.520	0.001 **
Make me feel beautiful	4.69 ± 0.48	4.31 ± 0.75	2.419	0.133
I think the content is readable	4.85 ± 0.55	4.62 ± 0.65	0.947	0.340
I am satisfied with the time taken to complete the task	4.85 ± 0.38	4.38 ± 0.65	4.909	0.036

** *p* < 0.01.

**Table 6:** Comfort and usability analysis of different sliding modes.

Dependent Variable	Sliding Up and Down (Average)	Sliding Left and Right (Average)
Comfort	4.75	4.285
Usability	4.75	4.565

**Table 7:** Normality test of key-style saccades.

Name	Sample Size	Average Value	Standard Deviation	Skewness	Kurtosis	Kolmogorov–Smirnov Test	
						Statistic D Value	*p*
Frequency	204	7.500	5.755	0.992	2.397	0.105	0.000 **

** *p* < 0.01.

**Table 8:** Homogeneous variance analysis of key-style saccades.

	Style (Standard Deviation)				F	*p*
	Type 1	Type 2	Type 3	Type 4		
Frequency	3.06	3.53	9.06	4.87	25.667	0.000 **

** *p* < 0.01.

**Table 9:** Analysis of variance for key-style saccades.

	Style (Mean ± SD)				F	*p*
	Type 1	Type 2	Type 3	Type 4		
Frequency	6.76 ± 3.06	8.570 ± 3.53	5.78 ± 9.06	8.39 ± 4.87	3.575	0.002 **

** *p* < 0.01.

**Table 10:** Data normality test for different styles of button fixation duration.

Name	Average Value	Standard Deviation	Skewness	Kurtosis	Shapro-Wilk Test	
					Statistic W Value	*p*
Fixation duration (proportion)	0.2237	0.745	0.043	0.030	0.932	0.407

*p* < 0.05, *p* < 0.01.

**Table 11:** Equal variance analysis of different styles of button fixation time.

	Style (Standard Deviation)				F	*p*
	Type 1	Type 2	Type 3	Type 4		
Fixation duration (proportion)	0.57	0.15	0.39	0.77	2.961	0.089

*p* < 0.05, *p* < 0.01.

**Table 12:** Analysis of variance for different styles of button fixation time.

	Style (Mean ± SD)				F	*p*
	Type 1	Type 2	Type 3	Type 4		
Fixation duration (proportion)	2.2292 ± 0.57	0.2242 ± 0.15	0.2142 ± 0.39	0.2269 ± 0.77	4.842	0.033 *

* *p* < 0.05, *p* < 0.01.

**Table 13:** Data normality test for different styles of button fixation duration.

Name	Average Value	Standard Deviation	Skewness	Kurtosis	Shapiro–Wilk Test	
					Statistic W Value	*p*
Fixation ratio	0.1933	11.135	−0.030	−1.524	0.904	0.180

*p* < 0.05, *p* < 0.01.

**Table 14:** Homogeneity of variance test for the proportion of key fixation points in different styles.

	Style (Standard Deviation)				F	*p*
	Type 1	Type 2	Type 3	Type 4		
Fixation ratio	12.88	15.80	10.32	12.53	0.284	0.836

*p* < 0.05, *p* < 0.01.

**Table 15:** Analysis of variance for different styles of button fixation time.

	Style (Mean ± SD)				F	*p*
	Type 1	Type 2	Type 3	Type 4		
Fixation ration	0.2033 ± 12.88	0.1947 ± 10.32	0.1914 ± 15.80	0.1839 ± 12.53	0.009	0.999

*p* < 0.05, *p* < 0.01.

**Table 16:** Normality test of user’s subjective preference data with different styles of keys.

Subject	Average Value	Standard Deviation	Skewness	Kurtosis
1	4.096	0.995	−0.695	−0.732
2	4.212	0.936	−1.041	0.216
3	4.154	0.958	−1.016	0.809
4	4.231	0.962	−1.312	1.539
5	4.423	1.036	−1.714	1.985
6	4.365	0.864	−1.748	2.779
7	4.346	0.947	−1.624	2.513
8	4.288	0.988	−1.408	1.625
9	4.269	0.952	−1.284	1.436

*p* < 0.05, *p* < 0.01.

**Table 17:** Homogeneity test of user’s subjective preferences data for different styles of keys.

Subject	Style (Standard Deviation)				F	*p*
	Type 1	Type 2	Type 3	Type 4		
1	1.00	1.24	0.91	0.65	2.381	0.081
2	1.12	1.12	0.76	0.65	1.264	0.297
3	1.26	1.04	0.80	0.66	2.054	0.119
4	1.26	0.99	0.80	0.63	1.205	0.318
5	1.41	1.14	0.77	0.28	10.877	0.000 **
6	1.34	0.75	0.66	0.52	3.674	0.018 *
7	1.34	1.04	0.66	0.51	3.455	0.024 *
8	1.38	1.04	0.77	0.51	4.469	0.008 **
9	1.19	0.95	0.87	0.63	1.077	0.368

* *p* < 0.05, ** *p* < 0.01.

**Table 18:** Analysis of variance of user’s subjective preference data with different styles of keys.

Subject	Style (Mean ± SD)				F	*p*
	Type 1	Type 2	Type 3	Type 4		
1	4.00 ± 1.00	3.77 ± 1.24	4.00 ± 0.91	4.62 ± 0.65	1.810	0.158
2	4.08 ± 1.12	4.08 ± 1.12	4.08 ± 0.76	4.62 ± 0.65	1.081	0.366
3	3.92 ± 1.26	4.08 ± 1.04	4.15 ± 0.80	4.46 ± 0.66	0.715	0.548
4	3.92 ± 1.26	4.15 ± 0.99	4.15 ± 0.80	4.69 ± 0.63	1.543	0.216
9	4.08 ± 1.19	3.92 ± 0.95	4.38 ± 0.87	4.69 ± 0.63	1.742	0.171

**Table 19:** Welch variance analysis of user’s subjective preference data with different styles of keys.

Subject	Style (Mean ± SD)				F	*p*
	Type 1	Type 2	Type 3	Type 4		
5	4.00 ± 1.41	4.15 ± 1.14	4.62 ± 0.77	4.92 ± 0.28	3.617	0.029 *
6	4.15 ± 1.34	4.31 ± 0.75	4.46 ± 0.66	4.54 ± 0.52	0.467	0.708
7	4.15 ± 1.34	4.08 ± 1.04	4.54 ± 0.66	4.62 ± 0.51	1.184	0.336
8	4.08 ± 1.38	4.08 ± 1.04	4.38 ± 0.77	4.62 ± 0.51	1.300	0.296

* *p* < 0.05, *p* < 0.01.

**Table 20:** Subjective evaluation scores of different styles of keys.

Dependent Variable	Style 1	Style 2	Style 3	Style 4
Comfort	4.04	3.925	4.04	4.62
Usability	4.037	4.142	4.38	4.64
Overall evaluation	4.08	3.92	4.38	4.69

**Table 21:** Analysis of variance in decision-making time for different button sizes.

	Dimensions (Mean ± SD)					F	*p*
	Type 1	Type 2	Type 3	Type 4	Type 5		
Duration(s)	7.94 ± 23.43	5.32 ± 2.47	6.30 ± 6.07	4.65 ± 2.79	6.12 ± 7.67	0.594	0.667

*p* < 0.05, *p* < 0.01.

**Table 22:** Normality test of saccade data of different size keys.

Name	Sample Size	Average Value	Standard Deviation	Skewness	Kurtosis	Kolmogorov-Smirnov Test	
						Statistic D Value	*p*
Frequency (n)	255	7.000	12.487	2.710	9.362	0.289	0.000 **
Frequency (n/min)	255	103.358	177.102	2.113	4.599	0.297	0.000 **

*p* < 0.05, ** *p* < 0.01.

**Table 23:** Homogeneous variance analysis of key saccade data of different sizes.

	Type (Standard Deviation)					F	*p*
	Type 1	Type 2	Type 3	Type 4	Type 5		
Frequency (n)	14.11	8.63	16.35	11.02	10.95	2.263	0.063
Frequency (n/min)	178.67	124.57	201.22	207.48	163.18	2.418	0.049 *

* *p* < 0.05, *p* < 0.01.

**Table 24:** Analysis of variance of eye movement times of different size keys.

	Type (Mean ± SD)					F	*p*
	Type 1	Type 2	Type 3	Type 4	Type 5		
Frequency	7.94 ± 14.11	4.55 ± 8.63	8.65 ± 16.35	6.90 ± 11.02	6.96 ± 10.95	0.784	0.537

*p* < 0.05, *p* < 0.01.

**Table 25:** Welch ANOVA of eye movement frequency of different size keys.

	Type (Mean ± SD)					F	*p*
	Type 1	Type 2	Type 3	Type 4	Type 5		
Frequency (n/min)	109.65 ± 178.67	69.04 ± 124.57	115.60 ± 201.22	121.29 ± 207.48	101.21 ± 163.18	0.979	0.422

*p* < 0.05, *p* < 0.01.

**Table 26:** The normality test of the proportion of the gaze duration of different sizes of buttons.

Name	Average Value	Standard Deviation	Skewness	Kurtosis	Shapiro–Wilk Test	
					Statistic W Value	*p*
Fixation duration ratio	0.1815	11.688	0.883	−0.028	0.916	0.167

*p* < 0.05, *p* < 0.01.

**Table 27:** Homogeneity of variance analysis for the proportion of fixation time on button of different sizes.

	Type (Mean ± SD)					F	*p*
	Type 1	Type 2	Type 3	Type 4	Type 5		
Fixation duration ratio	4.93	4.19	14.03	15.34	19.25	2.284	0.132

*p* < 0.05, *p* < 0.01.

**Table 28:** Analysis of variance for the proportion of fixation time on buttons of different sizes.

	Type (Mean ± SD)					F	*p*
	Type 1	Type 2	Type 3	Type 4	Type 5		
Fixation duration ratio	0.1370 ± 4.93	0.1985 ± 14.03	0.1333 ± 4.19	0.2171 ± 19.25	2213 ± 15.34	0.330	0.851

*p* < 0.05, *p* < 0.01.

**Table 29:** Data normality test for the proportion of button fixation points with different sizes.

Name	Average Value	Standard Deviation	Skewness	Kurtosis	Shapiro–Wilk Test	
					Statistic W Value	*p*
Fixation ratio	14.880	10.079	0.985	−0.206	0.875	0.040 *

* *p* < 0.05, *p* < 0.01.

**Table 30:** Homogeneity of variance analysis for the proportion of key fixation points of different sizes.

	Type (Standard Deviation)					F	*p*
	Type 1	Type 2	Type 3	Type 4	Type 5		
Fixation Ratio	2.13	3.48	13.12	9.89	14.44	2.367	0.123

*p* < 0.05, *p* < 0.01.

**Table 31:** Analysis of variance for the proportion of key fixation points of different sizes.

	Type (Mean ± SD)					F	*p*
	Type 1	Type 2	Type 3	Type 4	Type 5		
Fixation ratio	0.1112 ± 2.13	0.0612 ± 3.48	0.1977 ± 13.12	0.1874 ±9.89	0.186 ± 14.44	1.090	0.412

*p* < 0.05, *p* < 0.01.

## Data Availability

Not applicable.

## Abbreviations

The following abbreviations are used in this manuscript:

IoT	Internet of Things
SID	Scenario Interactive Design
SBD	Scenario-Based Design
AAL	Ambient Assisted Living
AALFI	Ambient Assisted Living Flexible Interface
AoI	Areas of Interest
SPSS	Statistical Product Service Solutions
